# Computationally Efficient Direction Finding for a Mixture of Circular and Strictly Noncircular Sources with Uniform Rectangular Arrays

**DOI:** 10.3390/s17061269

**Published:** 2017-06-02

**Authors:** Qing Wang, Xiaotian Zhu, Hua Chen, Laihua Wang, Weiqing Yan, Haoyu Fang

**Affiliations:** 1 School of Electrical Information Engineering, Tianjin University, 92 Weijin Road, Tianjin 300072, China; wangq@tju.edu.cn (Q.W.); zxtbronte@tju.edu.cn (X.Z.); fanghaoyuvl@tju.edu.cn (H.F.); 2 Faculty of Information Science and Engineering, Ningbo University, Ningbo 315211, China; 3 School of Software, Qufu Normal University, Qufu 273165, China; wlh@tju.edu.cn; 4 School of Computer and Control Engineering, Yantai University, Yantai 264005, China; wqyan@tju.edu.cn

**Keywords:** 2D DOA estimation, uniform rectangular arrays, noncircular signals, ROOT-MUSIC, theoretical error, low complexity

## Abstract

In this paper, a novel two-dimensional (2D) direction-of-arrival (DOA) estimation algorithm for the mixed circular and strictly noncircular sources is proposed. A general array model with a mixture of signals is firstly built based on uniform rectangular arrays (URAs), and then, the approach, which uses the rank-reduction-based ROOT-MUSIC, can solve 2D DOA estimation problem. Besides, the theoretical error of the proposed algorithm, a criterion of the performance for evaluation, is analyzed by the first-order Taylor expression using second-order statistics. As verified by the simulation results, a better DOA estimation performance and a lower computational complexity are achieved by the proposed algorithm than the existing methods resorting to the noncircularity of the incoming signals.

## 1. Introduction

Array signal processing has been widely used in the fields of sonar, radar, wireless communication, etc, and many excellent algorithms have been developed in the past few years. Especially the well-known multiple signal classification (MUSIC) algorithm has not only been applied in its time-reversal (TR) form to active location [1,2,3,4], but has also been widely used in direction-of-arrival (DOA) estimation (DOA is the research of passive location). In the last few years, DOA estimation became an important research branch of array signal processing [5,6,7]. Especially two-dimensional (2D) direction-of-arrival (DOA) estimation with different structured arrays, such as L-shaped uniform linear arrays (ULAs) [8,9,10], two-parallel ULAs [11,12,13], and uniform rectangular arrays (URAs) [14,15,16,17,18], has received much attention in past years. For URAs, the well-known multiple signal classification (MUSIC) algorithm can be used for 2D DOA estimation directly [16]; however, its computational complexity is very high. To overcome this problem, two efficient 2D DOA estimation methods have been proposed [17,18]. In [17], the reduced-dimension MUSIC algorithm was proposed, which reduced the computational complexity, and the 2D DOA estimation performance was very close to the 2D-MUSIC method. A preprocessing transformation matrix was introduced in [18], which transformed both the complex-valued covariance matrix and the complex-valued search vector into real-valued ones, then the 2D DOA estimation problem was decoupled into two successive real-valued one-dimensional (1D) DOA estimation problems with real-valued computations only. However, only the covariance matrix was considered, which characterizes the circular Gaussian distribution in the above 2D DOA methods.

In recent years, the issue of utilizing noncircular information has been attracting increasing attention. Abeida et al. presented a theoretical analysis of the resolution of the conventional and noncircular MUSIC algorithms and proved that the noncircular MUSIC algorithm for the threshold array signal-to-noise ratios are very sensitive to the noncircularity phase separation of the sources [19]. Pascal et al. introduced the optimal widely linear (WL) minimum variance distortionless response (MVDR) beamformer for the reception of an unknown signal of interest (SOI) corrupted by potentially second-order (SO) noncircular background noise and interference [20]. Wan et al. [21] proposed an algorithm that utilized the noncircular characteristic to solve the DOA estimation of coherently distributed sources. Because the covariance matrix and the elliptic covariance matrix (which is also named the complementary covariance or pseudo-covariance) are used in non-circular signals direction-finding method simultaneously, the DOA estimation performance can be improved greatly by exploiting the noncircularity information. Gan et al. studied the non-circular characteristics of the signals and proposed an automatically-paired 2D DOAs estimation method based on non-circular signals [22]. Based on strictly non-circular signals, Steinwandt et al. proposed the high-resolution R−D NCstandard ESPRIT and the R−D NC unitary ESPRIT algorithms, which are applicable to arbitrary shift-invariant R−D antenna arrays and do not require a centro-symmetric array structure [23]. In [24], an extended rank reduction (ERARE) method was introduced for noncircular sources based on two-parallel ULAs, which made the estimation more accurate than that in [12].

However, in modern wireless communications, a usual situation is that some users send circular signals such as quadrature phase shift keying (QPSK) signals, but others send non-circular ones such as binary phase shift keying (BPSK) signals. Therefore, the signals impinging to the array may be mixed ones. As for the mixed signals situation, a key issue is how to estimate and distinguish the circular and non-circular signals. In [25,26], the mixed signals situation issue has been studied, and the corresponding methods based on 1D arrays have been proposed. Gao et al. [25] combined the observed data and their conjugate counterparts to construct two 1D DOA estimators for detecting the circular and non-circular signals, but the DOA estimation performance of the method degraded seriously in the condition of a small separating angle. Liu et al. proposed an improved algorithm in [26], which detected the circular and non-circular signals by using the difference between the circularity of the mixed sources. However, to the best of our knowledge, few research works have reported the 2D DOA estimation problem for mixed circular and non-circular signals. In [27], based on two-parallel uniform linear arrays (ULAs), Chen proposed an effective algorithm that combined the rank reduction method and MUSIC algorithm to form four 1D DOA estimators for solving the 2D mixed signals situation. Nevertheless, the computational complexity of the algorithm in [27] is still high, and the theoretical performance analysis is never mentioned in this research.

In this paper, we study the 2D DOA estimation problem based on uniform rectangular arrays (URAs) using the rank-reduction-based ROOT-MUSIC method and the theoretical performance analysis of the proposed algorithm. Firstly, we establish an array model with mixed circular and noncircular sources with URAs; secondly, to avoid seeking the peak of the spectrum and reduce the computation load, a novel algorithm based on ROOT-MUSIC and the rank-reduction method is proposed to solve the 2D DOA estimation issue; finally, the theoretical error of the proposed algorithm is derived as a benchmark. Particularly, the paper mainly discusses the uncorrelated signals impinging upon the array. If we utilize some decorrelation methods such as the spatial smoothing technologies [28,29] or four-order cumulants-based Toeplitz matrices reconstruction (FOC-TMR) method [13] to preprocess the correlated signals and obtain the de-correlated matrices, the proposed method can also be generalized to the case of correlated sources.

The rest of this paper is organized as follows. Section 2 presents the array signal model. The description of the proposed algorithm is introduced in Section 3. The theoretical error analysis of the proposed algorithm is derived in Section 4. Finally, the simulation results are given in Section 5, and conclusions are drawn in Section 6.

Notations: ${\left(\cdot \right)}^{*}$ , ${\left(\cdot \right)}^{T}$ and ${\left(\cdot \right)}^{H}$ represent conjugation, transpose and conjugate transpose. E[·] is the expectation operation; diag(·) and blkdiag(·) stands for the diagonalization and block diagonalization operation, respectively; $I_{\omega }$ denotes the ω×ω dimensional identity matrix; det [·] indicates the determinant of a matrix; arg(·) is the phase angle operator. Expect $E_{n}$ , ${\hat{E}}_{n}$ , $E_{n1}$ , ${\hat{E}}_{n1}$ , $E_{n2}$ and ${\hat{E}}_{n2}$ representing the corresponding noise subspace; other variables have index *n* such as ${\left(\cdot \right)}_{n}$ , which denote ones related to the non-circular signal. ${\left(\cdot \right)}_{c}$ indicates a variable associated with the circular signal.

## 2. Problem Formulation

**Figure 1:**
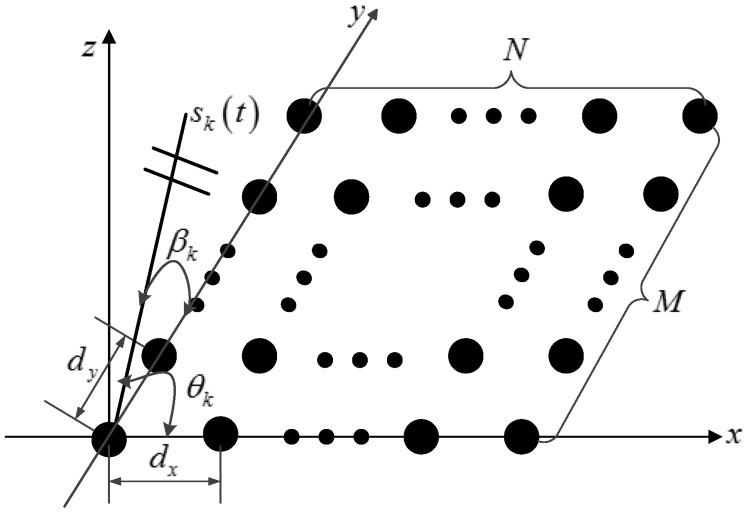
Geometry of a uniform rectangular array (URA) with N×M sensors.

In this paper, we suppose that the number of signals is known or is estimated by the existing number detection technique in advance [30]. As illustrated in Figure 1, consider that *K* uncorrelated far-field narrowband signals $s_{k}\left(t\right)\;$ (*k* = 1, 2, ..., *K*) impinging upon the array with $K_{n}$ noncircular signals $s_{n,k_{n}}\left(t\right)\;(k_{n}=1,\;2,\;...,\;K_{n})$ and $K_{c}$ circular signals $s_{c,k_{c}}\left(t\right)\;(k_{c}=1,\;2,\;...,\;K_{c})$ , from directions $\left(\theta_{k},\beta_{k}\right)$ , k=1,2,...,K , where $K=K_{n}+K_{c}$ . The array is composed of uniform rectangular arrays (URAs) with N×M omnidirectional sensors spaced by $d_{x}$ in the x-axis direction and $d_{y}$ in the y-axis direction. λ is the wavelength of the incident waves, and $d_{x}$ = $d_{y}$ = λ/2 . The additive noises of the URAs are circular Gaussian with zero mean and variance $\sigma^{2}$ , which are uncorrelated with the impinging signals. The received data vectors of the URAs at sample *t* can be expressed as:

(1)[H]x1(t)=AG1s˜(t)+n1(t)(2)x2(t)=AG2s˜(t)+n2(t)⋮(3)xN(t)=AGNs˜(t)+nN(t)

where $x_{1}\left(t\right)=\left[x_{11}\left(t\right),\;x_{12}\left(t\right),\;\ldots \;,x_{1M}\left(t\right)\right]$ , $x_{2}\left(t\right)=\left[x_{21}\left(t\right),\;x_{22}\left(t\right),\;\ldots ,\;x_{2M}\left(t\right)\right]$ , ... , $x_{N}\left(t\right)=\left[x_{N1}\left(t\right),\;x_{N2}\left(t\right),\;\ldots ,\;x_{NM}\left(t\right)\right]$ . $A=\left[a\left(\theta_{1}\right),\;a\left(\theta_{2}\right),\;\ldots ,\;a\left(\theta_{K}\right)\right]$ is the steering matrix with each column denoted by $a\left(\theta_{k}\right)={\left[a_{1}\left(\theta_{k}\right),\;\ldots ,\;a_{M}\left(\theta_{k}\right)\right]}^{T}$ , k=1,2,…,K , with $a_{i}\left(\theta_{k}\right)=e^{-j\frac{2\pi }{\lambda }d_{x}\left(i-1\right)cos\theta_{k}}$ , i=1,2,…,M . $G_{\zeta }$ , ζ=1,2,…,N , is termed the steering element matrix given by $G_{\zeta }=diag\left[\upsilon_{\zeta }\left(\beta_{1}\right),\;\upsilon_{\zeta }\left(\beta_{2}\right),\;\ldots ,\;\upsilon_{\zeta }\left(\beta_{K}\right)\right]$ with $\upsilon_{\zeta }\left(\beta_{k}\right)=e^{j\frac{2\pi }{\lambda }\left(\zeta -1\right)d_{y}cos\beta_{k}}$ . $n_{1}\left(t\right)={\left[n_{1,1}\left(t\right),\;n_{1,2}\left(t\right),\;\ldots ,\;n_{1,M}\left(t\right)\right]}^{T}$ , $n_{2}\left(t\right)={\left[n_{2,1}\left(t\right),\;n_{2,2}\left(t\right),\;\ldots ,\;n_{2,M}\left(t\right)\right]}^{T}$ , ... , and $n_{N}\left(t\right)={\left[n_{N,1}\left(t\right),\;n_{N,2}\left(t\right),\;\ldots ,\;n_{N,M}\left(t\right)\right]}^{T}$ indicate the circular Gaussian noise vectors of the URAs, respectively. $\tilde{s}\left(t\right)$ is the mixed signal vector, which can be denoted as $\tilde{s}\left(t\right)={\left[s_{n,1}\left(t\right),\;\ldots ,\;s_{n,K_{n}}\left(t\right),\;s_{c,1}\left(t\right),\;\ldots ,\;s_{c,K_{c}}\left(t\right)\right]}^{T}$ , and we can see that there are $K_{n}$ noncircular and $K_{c}$ circular signals in it.

In practice, non-circularity and circularity are important properties of random variables; their concept directly comes from the geometrical interpretation of complex random variable. The signal would be called the circular source if its statistical characteristic has the rotational invariance characteristic, otherwise, it would be called the noncircular source. The work in [31] introduces the circularity and noncircularity in detail. Based on this, we only consider the rotational invariance characteristic of the first- and second-order statistical properties of the sources. For a complex random signal $s_{k}$ , k=1,2,...,K , we defined $E[s_{k}]$ , $E[s_{k}{s_{k}}^{*}]$ and $E[{s_{k}}^{2}]$ as the mean, the covariance and the elliptic covariance of the signal $s_{k}$ , respectively. If the source’s first- and second-order statistical properties are rotational invariant for an arbitrary phase $\varphi_{k}$ as follows:

(4) $$E\left[s_{k}e^{j\varphi }\right]=E\left[s_{k}\right]$$

(5) $$E\left[s_{k}e^{j\varphi_{k}}{\left(s_{k}e^{j\varphi_{k}}\right)}^{*}\right]=E\left[s_{k}{s_{k}}^{*}\right]$$

(6) $$E\left[s_{k}e^{j\varphi_{k}}\cdot s_{k}e^{j\varphi_{k}}\right]=E\left[{s_{k}}^{2}\right]$$

The signal $s_{k}$ will be called the circular source. Conversely, the signal $s_{k}$ will be noncircular if the first- and second-order statistical properties are not rotational invariant.

Beyond that, reference [32] proposes a model to describe signal sources with arbitrary second-order non-circularity. The source $s_{k}$ is defined as follows:

(7) $$s_{k}=e^{j\varphi_{k}}\left(\sqrt{\frac{1+\eta_{k}}{2}}s_{I_{k}}+j\sqrt{\frac{1-\eta_{k}}{2}}s_{Q_{k}}\right),k=1,2,...K$$

where $\varphi_{k}$ is the rotation phase, $\eta_{k}\;\left(0\leq \eta_{k}\leq 1\right)$ denotes the non-circularity coefficient and $s_{I_{k}}$ and $s_{Q_{k}}$ represent the in-phase and quadrature components of the complex signal $s_{k}$ , respectively. Therefore, $s_{k}$ will represent a circular source if $\eta_{k}=0$ , where the rotation phase $\varphi_{k}$ is irrelevant and undetermined. If $\eta_{k}=1$ , $s_{k}$ will represent a strictly non-circular signal.

Based on the above research, we defined the strictly non-circular signal as $s_{n,k_{n}}\left(t\right)=b_{n,k_{n}}{\bar{s}}_{n,k_{n}}\left(t\right)$ , where ${\bar{s}}_{n,k_{n}}\left(t\right)$ is a real signal and $b_{n,k_{n}}=e^{j\varphi_{k_{n}}}\left(k_{n}=1,\;2,\;\ldots ,\;K_{n}\right)$ is an arbitrary phase shift for the signal. Due to the phase information of circular source being irrelevant, it can be represented as $s_{c,k_{c}}\left(t\right),k_{c}=1,\;2,\;\ldots ,\;K_{c}$ . Therefore, the mixed source signal vector can be modeled as $\tilde{s}\left(t\right)=\mathrm{Bs}\left(t\right)$ . B can be expressed as:

(8) $$B=diag\left[b_{n,1},\;\ldots ,\;b_{n,\;K_{n}},\;\underset{K_{c}}{\underset{︸}{1,\;\ldots ,\;1}}\right]=\left[\begin{array}{cc} B_{1} & 0\\ 0 & B_{2} \end{array}\right]$$

where $B_{1}=diag\left[b_{n,1},\;\ldots ,\;b_{n,\;K_{n}}\right]$ , $B_{2}=I_{K_{c}}$ , and s(t) is denoted as:

(9) $$s\left(t\right)={\left[{\bar{s}}_{n,1}\left(t\right),\;\ldots ,\;{\bar{s}}_{n,K_{n}}\left(t\right),s_{c,1}\left(t\right),\;\ldots ,\;s_{c,K_{c}}\left(t\right)\right]}^{T}$$

A new data vector f(t) is defined by concatenating the received data vectors $x_{1}\left(t\right)$ , $x_{2}\left(t\right)$ , ... , and $x_{N}\left(t\right)$ as follows:

(10) $$\begin{array}{cc} f\left(t\right) & =\left[\begin{array}{c} x_{1}\left(t\right)\\ x_{2}\left(t\right)\\ \vdots \\ x_{N}\left(t\right) \end{array}\right]=\left[\begin{array}{c} A\left(\theta \right)G_{1}\left(\beta \right)\\ A\left(\theta \right)G_{2}\left(\beta \right)\\ \vdots \\ A\left(\theta \right)G_{N}\left(\beta \right) \end{array}\right]\mathrm{Bs}\left(t\right)+\left[\begin{array}{c} n_{1}\left(t\right)\\ n_{2}\left(t\right)\\ \vdots \\ n_{N}\left(t\right) \end{array}\right]\\ & =C\left(\theta ,\;\beta \right)\mathrm{Bs}\left(t\right)+n\left(t\right) \end{array}$$

where C(θ,β) is the extend steering vector, and:

(11) $$C=[C_{1}\left(\theta_{n},\;\beta_{n}\right)C_{2}\left(\theta_{c},\;\beta_{c}\right)]$$

In order to simplify the notation, the pair of angles (θ,β) and *t* is omitted. In Equation (11),

(12) $$\begin{array}{cc} C_{1} & =\left[\begin{array}{ccc} \begin{array}{c} a\left(\theta_{n,1}\right)\upsilon_{1}\left(\beta_{n,1}\right)\\ a\left(\theta_{n,1}\right)\upsilon_{2}\left(\beta_{n,1}\right)\\ \vdots \\ a\left(\theta_{n,1}\right)\upsilon_{N}\left(\beta_{n,1}\right) \end{array} & \cdots & \begin{array}{c} a\left(\theta_{n,K_{n}}\right)\upsilon_{1}\left(\beta_{n,K_{n}}\right)\\ a\left(\theta_{n,K_{n}}\right)\upsilon_{2}\left(\beta_{n,K_{n}}\right)\\ \vdots \\ a\left(\theta_{n,K_{n}}\right)\upsilon_{N}\left(\beta_{n,K_{n}}\right) \end{array} \end{array}\right]\;\\ & =\left[\begin{array}{ccc} \begin{array}{c} a_{n,1}\upsilon_{1_{n,1}}\\ a_{n,1}\upsilon_{2_{n,1}}\\ \vdots \\ a_{n,1}\upsilon_{N_{n,1}} \end{array} & \cdots & \begin{array}{c} a_{n,K_{n}}\upsilon_{1_{n,K_{n}}}\\ a_{n,K_{n}}\upsilon_{2_{n,K_{n}}}\\ \vdots \\ a_{n,K_{n}}\upsilon_{N_{n,K_{n}}} \end{array} \end{array}\right] \end{array}$$

(13) $$\begin{array}{cc} C_{2} & =\left[\begin{array}{ccc} \begin{array}{c} a\left(\theta_{c,1}\right)\upsilon_{1}\left(\beta_{c,1}\right)\\ a\left(\theta_{c,1}\right)\upsilon_{2}\left(\beta_{c,1}\right)\\ \vdots \\ a\left(\theta_{c,1}\right)\upsilon_{N}\left(\beta_{c,1}\right) \end{array} & \cdots & \begin{array}{c} a\left(\theta_{c,K_{c}}\right)\upsilon_{1}\left(\beta_{c,K_{c}}\right)\\ a\left(\theta_{c,K_{c}}\right)\upsilon_{2}\left(\beta_{c,K_{c}}\right)\\ \vdots \\ a\left(\theta_{c,K_{c}}\right)\upsilon_{N}\left(\beta_{c,K_{c}}\right) \end{array} \end{array}\right]\;\\ & =\left[\begin{array}{ccc} \begin{array}{c} a_{c,1}\upsilon_{1_{c,1}}\\ a_{c,1}\upsilon_{2_{c,1}}\\ \vdots \\ a_{c,1}\upsilon_{N_{c,1}} \end{array} & \cdots & \begin{array}{c} a_{c,K_{c}}\upsilon_{1_{c,K_{c}}}\\ a_{c,K_{c}}\upsilon_{2_{c,K_{c}}}\\ \vdots \\ a_{c,K_{c}}\upsilon_{N_{c,K_{c}}} \end{array} \end{array}\right] \end{array}$$

$C_{1}$ is a $NM\times K_{n}$ matrix of noncircular signals and $C_{2}$ is a $NM\times K_{c}$ matrix of circular signals.

As a classical procedure for existing noncircular DOA estimation algorithms [27,33,34], we can construct a new augmented data matrix $\overset{⌣}{\;f}$ by combining the vector f and its conjugate counterpart $f^{*}$ as follows:

(14) $$\overset{⌣}{\;f}=\left[\begin{array}{c} f\\ f^{*} \end{array}\right]=\left[\begin{array}{c} \mathrm{CBs}\\ C^{*}B^{*}s^{*} \end{array}\right]+\left[\begin{array}{c} n\\ n^{*} \end{array}\right]=\overset{⌣}{\;C}\overset{⌣}{\;s}+\overset{⌣}{\;n}$$

The procedure can extend the array virtually and enlarge the aperture of the array antenna, and the estimating precision of DOA can be improved by utilizing the noncircularity of signals. In Equation (14), $\overset{⌣}{\;C}$ is a $2NM\times (K_{n}\;+\;2K_{c})$ matrix, which contains the new steering vectors of the impinging sources. $\overset{⌣\;}{\;s}\;$ is a $(K_{n}\;+\;2K_{c})\times 1$ matrix of signals.

(15) $$\overset{⌣}{\;C}=[{\overset{⌣}{\;c}}_{n,1},\;\ldots ,\;{\overset{⌣}{\;c}}_{n,K_{n}},\;{\overset{⌣}{\;c}}_{c,1},\;\ldots ,{\overset{⌣}{\;c}}_{c,K_{c}}]$$

where ${\overset{⌣}{\;c}}_{n,k_{n}}$ and ${\overset{⌣}{\;c}}_{c,k_{c}}$ are the new steering vectors of non-circular signals and circular signals, respectively.

(16) $${\overset{⌣}{\;c}}_{n,k_{n}}=\left[\begin{array}{c} b_{n,k_{n}}\left(\begin{array}{c} a_{n,k_{n}}\upsilon_{1_{n,k_{n}}}\\ a_{n,k_{n}}\upsilon_{2_{n,k_{n}}}\\ \vdots \\ a_{n,k_{n}}\upsilon_{N_{n,k_{n}}} \end{array}\right)\\ b_{n,k_{n}}^{*}\left(\begin{array}{c} a_{n,k_{n}}^{*}\upsilon_{1_{n,k_{n}}}^{*}\\ a_{n,k_{n}}^{*}\upsilon_{2_{n,k_{n}}}^{*}\\ \vdots \\ a_{n,k_{n}}^{*}\upsilon_{N_{n,k_{n}}}^{*} \end{array}\right) \end{array}\right]$$

$k_{n}=1,\;2,\;\ldots ,\;K_{n}$ , which is a 2NM×1 vector,

(17) $${\overset{⌣}{\;c}}_{c,k_{c}}=\left[\begin{array}{cc} \begin{array}{c} a_{c,k_{c}}\upsilon_{1_{c,k_{c}}}\\ a_{c,k_{c}}\upsilon_{2_{c,k_{c}}}\\ \vdots \\ a_{c,k_{c}}\upsilon_{N_{c,k_{c}}} \end{array} & \begin{array}{c} 0_{M\times 1}\\ 0_{M\times 1}\\ \vdots \\ 0_{M\times 1} \end{array}\\ \begin{array}{c} 0_{M\times 1}\\ 0_{M\times 1}\\ \vdots \\ 0_{M\times 1} \end{array} & \begin{array}{c} a_{c,k_{c}}^{*}\upsilon_{1_{c,k_{c}}}^{*}\\ a_{c,k_{c}}^{*}\upsilon_{2_{c,k_{c}}}^{*}\\ \vdots \\ a_{c,k_{c}}^{*}\upsilon_{N_{c,k_{c}}}^{*} \end{array} \end{array}\right]$$

$k_{c}=1,\;2,\;\ldots ,\;K_{c}$ , which is a 2NM×2 matrix,

(18) $$\begin{array}{c} \overset{⌣}{\;s}\left(t\right)=[s_{n,1}\left(t\right),\;\cdots ,\;s_{n,K_{n}}\left(t\right),\;s_{c,1}\left(t\right),\;s_{c,1}^{*}\left(t\right),\;\cdots ,\;s_{c,K_{c}}\left(t\right),s_{c,K_{c}}^{*}\left(t\right){]}^{T} \end{array}$$

which is a $(K_{n}\;+\;2K_{c})\times 1$ matrix of signals, and:

(19) $$\overset{⌣}{\;n}=\left[\begin{array}{c} n\\ n^{*} \end{array}\right]$$

which is a 2NM×1 vector of noise.

The covariance matrix of $\overset{⌣}{\;f}$ is calculated by:

(20) $$\overset{⌣}{\;R}=E\left[\overset{⌣}{\;f}{\overset{⌣}{\;f}}^{H}\right]=\overset{⌣}{\;C}{\overset{⌣}{\;R}}_{s}{\overset{⌣}{\;C}}^{H}+\sigma^{2}I_{2NM}$$

where ${\overset{⌣}{\;R}}_{s}=E\left[\overset{⌣}{\;s}{\overset{⌣}{\;s}}^{H}\right]$ is the covariance matrix of $\overset{⌣}{\;s}$ . ${\overset{⌣}{\;R}}_{s}$ is a full-rank matrix, since the incident signals are uncorrelated with each other. Then, the eigenvalue decomposition of $\overset{⌣}{\;R}$ is:

(21) $$\overset{⌣}{\;R}=E_{s}\Sigma_{s}E_{s}^{H}+E_{n}\Sigma_{n}E_{n}^{H}$$

where the $2NM\times (K_{n}\;+\;2K_{c})$ matrix $E_{s}$ and the $2NM\times (2NM-K_{n}-2K_{c})$ matrix $E_{n}$ are the signal subspace and noise subspace, respectively. The $\left(K_{n}+2K_{c}\right)\times \left(K_{n}+2K_{c}\right)$ matrix $\Sigma_{s}=diag\left(\lambda_{1},\;\lambda_{2},\;\ldots ,\;\lambda_{K}\right)$ and the $\left(2NM-K_{n}-2K_{c}\right)\times \left(2NM-K_{n}-2K_{c}\right)$ matrix $\Sigma_{n}=diag\left(\lambda_{K+1},\;\lambda_{K+2},\;\ldots ,\;\lambda_{2NM}\right)$ are diagonal matrices, where $\lambda_{1}\geq \lambda_{2}\geq \cdots \geq \lambda_{K}>\lambda_{K+1}=\cdots =\lambda_{2NM}=\sigma^{2}$ are the eigenvalues of $\overset{⌣}{\;R}$ .

**Remark** **1.** *In practice, the available observed data are finite. Thus, $\overset{⌣}{\;R}$ can be approximated by:*

(22) $$\hat{\overset{⌣}{\;R}}=\frac{1}{L}\sum_{t=1}^{L}\overset{⌣}{\;f}(t){\overset{⌣}{\;f}}^{H}\left(t\right)$$

*where L is the number of available data snapshots. Then, the eigenvalue decomposition of* $\hat{\overset{⌣}{\;R}}$ *is:*

(23) $$\hat{\overset{⌣}{\;R}}={\hat{E}}_{s}{\hat{\Sigma }}_{s}{\hat{E}}_{s}^{H}+{\hat{E}}_{n}{\hat{\Sigma }}_{n}{\hat{E}}_{n}^{H}$$

*therefore, the noise subspace* $E_{n}$ *and signal subspace* $E_{s}$ *can be approximated by* ${\hat{E}}_{n}$ *and* ${\hat{E}}_{s}$ *, respectively.*

## 3. The Proposed Algorithm

In this section, we propose a 2D DOA estimation algorithm to solve the problem of estimating and distinguishing the mixed signals that are circular and non-circular in detail. Firstly, the method for estimating the mixed sources that are circular and noncircular is proposed; secondly, the algorithm, which only detects the circular signals, is proposed; and finally, we study how to distinguish these two kinds of sources.

Because both $\overset{⌣}{\;C}$ and $E_{s}$ have the same signal subspace, orthogonal to the noise subspace spanned by the matrix $E_{n}$ , we devise estimators to obtain the 2D DOAs of noncircular and circular signals using the rank-reduction-based Root-MUSIC method.

### 3.1. 2D DOA Estimation for Non-Circular Sources

Because the noise subspace $E_{n}$ is orthogonal to ${\overset{⌣}{\;c}}_{n,k_{n}}$ (the steering vectors of non-circular signals), the following equation can be obtained directly:

(24) $$E_{n}^{H}{\overset{⌣}{\;c}}_{n,k_{n}}=0$$

Then, together with Equations (16) and (24), we can get the following equation:

(25) $$\begin{array}{cc} E_{n}^{H}{\overset{⌣}{\;c}}_{n,k_{n}}\; & =\;E_{n}^{H}\left[\begin{array}{c} b_{n,k_{n}}\left(\begin{array}{c} a_{n,k_{n}}\upsilon_{1_{{}_{n,k_{n}}}}\\ a_{n,k_{n}}\upsilon_{2_{{}_{n,k_{n}}}}\\ \vdots \\ a_{n,k_{n}}\upsilon_{N_{{}_{n,k_{n}}}} \end{array}\right)\\ b_{n,k_{n}}^{*}\left(\begin{array}{c} {a^{*}}_{n,k_{n}}\upsilon_{1_{n,k_{n}}}^{*}\\ {a^{*}}_{n,k_{n}}\upsilon_{2_{n,k_{n}}}^{*}\\ \vdots \\ {a^{*}}_{n,k_{n}}\upsilon_{N_{n,k_{n}}}^{*} \end{array}\right) \end{array}\right]\\ & =\;E_{n}^{H}blkdiag\left[a_{n,k_{n}},\;a_{n,k_{n}},\;\ldots ,\;a_{n,k_{n}},\;a_{n,k_{n}}^{*},\;a_{n,k_{n}}^{*},\;\ldots ,\;a_{n,k_{n}}^{*}\right].\\ & \;\left[\begin{array}{cc} \upsilon_{1_{{}_{n,k_{n}}}} & 0\\ \begin{array}{c} \upsilon_{2_{{}_{n,k_{n}}}}\\ \vdots \\ \upsilon_{N_{{}_{n,k_{n}}}} \end{array} & \begin{array}{c} 0\\ \vdots \\ 0 \end{array}\\ 0 & \upsilon_{1_{n,k_{n}}}^{*}\\ \begin{array}{c} 0\\ \vdots \\ 0 \end{array} & \begin{array}{c} \upsilon_{2_{n,k_{n}}}^{*}\\ \vdots \\ \upsilon_{N_{n,k_{n}}}^{*} \end{array} \end{array}\right]\left[\begin{array}{c} b_{n,k_{n}}\\ b_{n,k_{n}}^{*} \end{array}\right]=0 \end{array}$$

Defining a vector $p_{n}\left(l\right)={\left[1,\;l,\;...,\;l^{M-1}\right]}^{T}$ , which is only related to *l*, where l=e−j(2π(2πλλ)dxcosθn,kn . Therefore, we can obtain the formula $p_{n}\left(l\right)=a_{n,k_{n}}$ and $p_{n}\left(l^{-1}\right)={a^{*}}_{n,k_{n}}$ , then define a 2NM×2N matrix Ω (*l*), which is only related to *l*,

(26) $$\begin{array}{c} \Omega \left(l\right)\;=blkdiag[p_{n}\left(l\right),\;p_{n}\left(l\right),\;\cdots ,\;p_{n}\left(l\right),\;p_{n}\left(l^{-1}\right),\;p_{n}\left(l^{-1}\right),\;\cdots ,\;p_{n}\left(l^{-1}\right)]\;\;\\ =blkdiag\left[a_{n,k_{n}},\;a_{n,k_{n}},\;\cdots ,\;a_{n,k_{n}},\;a_{n,k_{n}}^{*},\;a_{n,k_{n}}^{*},\;\cdots ,a_{n,k_{n}}^{*}\right] \end{array}$$

and define a 2N×2N matrix:

(27) $$Q_{n}\left(l\right)=l^{M-1}\Omega^{T}\left(l^{-1}\right)E_{n}E_{n}^{H}l^{M-1}\Omega \left(l\right)$$

Note that if $(2NM-K_{n}-2K_{c})\geq 2N$ and θ is not the true angle of the non-circular signal, $Q_{n}\left(l\right)$ is of full rank because in this case, the column rank of $E_{n}$ is not less than 2N . Then, Equation (25) holds true only when θ equals the true angle of the signal ( $Q_{n}\left(l\right)$ drops rank). Since the covariance matrix of $\overset{⌣}{\;f}$ is obtained from a finite number of samples, the reduction of the rank of $Q_{n}\left(l\right)$ can roughly be replaced by the minimum of the determinant of $Q_{n}\left(l\right)$ . Therefore, we get the estimator of θ about non-circular signals as follows:

(28) $$f_{n}\left(l\right)=\det\left(Q_{n}\left(l\right)\right)=\det\left(l^{M-1}\Omega^{T}\left(l^{-1}\right)E_{n}E_{n}^{H}l^{M-1}\Omega \left(l\right)\right)$$

Notice that $f_{n}\left(l\right)$ is a 2×3N(M−1) order polynomial, showing that there are 3N(M−1) pairs of conjugated roots. The estimates $K_{n}$ signal DOAs of θ can be obtained by finding the closest roots of the unit circle, which are given by:

(29) $${\hat{\theta }}_{k_{n}}=\arccos\left[-\frac{\lambda }{2\pi d_{x}}\arg\left(l_{k_{n}}\right)\right],k_{n}=1,...,K_{n}$$

where $l_{k_{n}},k_{n}=1,\;...,\;K_{n}$ are the roots closest to the unit circle. Then, we take the estimated ${\hat{\theta }}_{k_{n}}$ of non-circular source into Equation (25) to get the estimator of β :

(30) $$f_{n}^{\prime }\left(u\right)=\det\left(Q_{n}^{\prime }\left(u\right)\right)$$

where:

(31) $$Q_{n}^{\prime }\left(u\right)=u^{\left(N-1\right)}\Theta^{T}\left(u^{-1}\right)\Omega^{T}\left(l^{-1}\right)E_{n}E_{n}^{H}\Omega \left(l\right)u^{\left(N-1\right)}\Theta \left(u\right)$$

u=ej(2π(2πλλ)dycosβn,kn ; it means that $\upsilon_{m_{n,k_{n}}}=u^{m-1},\;m=1,\;\cdots N$ and:

(32) $$\Theta \left(u\right)=\left[\begin{array}{cc} 1 & 0\\ \begin{array}{c} u\\ \vdots \\ u^{N-1} \end{array} & \begin{array}{c} 0\\ \vdots \\ 0 \end{array}\\ 0 & 1\\ \begin{array}{c} 0\\ \vdots \\ 0 \end{array} & \begin{array}{c} u^{*}\\ \vdots \\ {u^{*}}^{N-1} \end{array} \end{array}\right]=\left[\begin{array}{cc} \upsilon_{1_{n,k_{n}}}\left(\beta \right) & 0\\ \begin{array}{c} \upsilon_{2_{n,k_{n}}}\left(\beta \right)\\ \vdots \\ \upsilon_{N_{n,k_{n}}}\left(\beta \right) \end{array} & \begin{array}{c} 0\\ \vdots \\ 0 \end{array}\\ 0 & \upsilon_{1_{n,k_{n}}}^{*}\left(\beta \right)\\ \begin{array}{c} 0\\ \vdots \\ 0 \end{array} & \begin{array}{c} \upsilon_{2_{n,k_{n}}}^{*}\left(\beta \right)\\ \vdots \\ \upsilon_{N_{n,k_{n}}}^{*}\left(\beta \right) \end{array} \end{array}\right]$$

To achieve the estimate of β , we need to solve the roots of $f_{n}^{\prime }\left(u\right)$ . Due to $f_{n}^{\prime }\left(u\right)$ being a 2×3(N−1) -order polynomial, it means there are 3(N−1) pairs of conjugated roots. The estimate of β can be obtained by finding the closest roots of the unit circle; in this way, we can automatically pair the closest roots $u_{1},\;u_{2},\;...,\;u_{K_{n}}$ corresponding to ${\hat{\theta }}_{1},\;{\hat{\theta }}_{2},\;\ldots ,\;{\hat{\theta }}_{K_{n}}$ , respectively, and the DOAs of β are given by:

(33) $${\hat{\beta }}_{k_{n}}=\arccos\left[\frac{\lambda }{2\pi d_{y}}\arg\left(u_{k_{n}}\right)\right],\;k_{n}=1,\;...,\;K_{n}$$

where $u_{k_{n}},k_{n}=1,\;...,\;K_{n}$ are the roots closest to the unit circle, respectively.

### 3.2. 2D DOA Estimation for Circular Sources

Because the noise subspace $E_{n}$ is also orthogonal to ${\overset{⌣}{\;c}}_{c,k_{c}}$ (the steering vectors of circular signals), we can get the equation as follows:

(34) $$E_{n}^{H}{\overset{⌣}{\;c}}_{c,k_{c}}=E_{n}^{H}\left[\begin{array}{cc} a_{c,k_{c}}\upsilon_{1_{{}_{c,k_{c}}}} & 0_{M\times 1}\\ a_{c,k_{c}}\upsilon_{2_{c,k_{c}}} & 0_{M\times 1}\\ \vdots & \vdots \\ a_{c,k_{c}}\upsilon_{N_{{}_{c,k_{c}}}} & 0_{M\times 1}\\ 0_{M\times 1} & a_{c,k_{c}}^{*}{\upsilon^{*}}_{1_{{}_{c,k_{c}}}}\\ 0_{M\times 1} & a_{c,k_{c}}^{*}{\upsilon^{*}}_{2_{{}_{c,k_{c}}}}\\ \vdots & \vdots \\ 0_{M\times 1} & a_{c,k_{c}}^{*}{\upsilon^{*}}_{N_{{}_{c,k_{c}}}} \end{array}\right]=0$$

from Equation (34), we can get:

(35) $$E_{n}^{H}\left[\begin{array}{c} a_{c,k_{c}}\upsilon_{1_{{}_{c,k_{c}}}}\\ a_{c,k_{c}}\upsilon_{2_{c,k_{c}}}\\ \vdots \\ a_{c,k_{c}}\upsilon_{N_{{}_{c,k_{c}}}}\\ 0_{M\times 1}\\ 0_{M\times 1}\\ \vdots \\ 0_{M\times 1} \end{array}\right]=0$$

(36) $$E_{n}^{H}\left[\begin{array}{c} 0_{M\times 1}\\ 0_{M\times 1}\\ \vdots \\ 0_{M\times 1}\\ {a^{*}}_{c,k_{c}}{\upsilon^{*}}_{1_{{}_{c,k_{c}}}}\\ {a^{*}}_{c,k_{c}}{\upsilon^{*}}_{2_{c,k_{c}}}\\ \vdots \\ {a^{*}}_{c,k_{c}}{\upsilon^{*}}_{N_{{}_{c,k_{c}}}} \end{array}\right]=0$$

Partitioning the noise subspace $E_{n}$ into $E_{n}=\left[\begin{array}{c} E_{n1}\\ E_{n2} \end{array}\right]$ , where $E_{n1}$ and $E_{n2}$ are two submatrices of the same size $NM\times (2NM-K_{n}-2K_{c})$ , Equations (35) and (36) can be changed to:

(37) $$\begin{array}{c} \;\;{\left[\begin{array}{c} a_{c,k_{c}}\upsilon_{1_{c,k_{c}}}\\ \begin{array}{c} a_{c,k_{c}}\upsilon_{2_{c,k_{c}}}\\ \vdots \\ a_{c,k_{c}}\upsilon_{N_{c,k_{c}}} \end{array} \end{array}\right]}^{H}E_{n1}E_{n1}^{H}\left[\begin{array}{c} a_{c,k_{c}}\upsilon_{1_{c,k_{c}}}\\ \begin{array}{c} a_{c,k_{c}}\upsilon_{2_{c,k_{c}}}\\ \vdots \\ a_{c,k_{c}}\upsilon_{N_{c,k_{c}}} \end{array} \end{array}\right]\;\\ \;={\left[\begin{array}{c} \upsilon_{1_{c,k_{c}}}\\ \begin{array}{c} \upsilon_{2_{c,k_{c}}}\\ \vdots \\ \upsilon_{N_{c,k_{c}}} \end{array} \end{array}\right]}^{H}blkdiag{\left[a_{c,k_{c}},\;a_{c,k_{c}},\;\cdots ,\;a_{c,k_{c}}\right]}^{H}.\\ E_{n1}E_{n1}^{H}blkdiag\left[a_{c,k_{c}},\;a_{c,k_{c}},\;\cdots ,\;a_{c,k_{c}}\right]\left[\begin{array}{c} \upsilon_{1_{c,k_{c}}}\\ \begin{array}{c} \upsilon_{2_{c,k_{c}}}\\ \vdots \\ \upsilon_{N_{c,k_{c}}} \end{array} \end{array}\right]=0 \end{array}$$

(38) $$\begin{array}{c} \;\;{\left[\begin{array}{c} {a^{*}}_{c,k_{c}}{\upsilon^{*}}_{1_{c,k_{c}}}\\ \begin{array}{c} {a^{*}}_{c,k_{c}}{\upsilon^{*}}_{2_{c,k_{c}}}\\ \vdots \\ {a^{*}}_{c,k_{c}}{\upsilon^{*}}_{N_{c,k_{c}}} \end{array} \end{array}\right]}^{H}E_{n2}E_{n2}^{H}\left[\begin{array}{c} {a^{*}}_{c,k_{c}}{\upsilon^{*}}_{1_{c,k_{c}}}\\ \begin{array}{c} {a^{*}}_{c,k_{c}}{\upsilon^{*}}_{2_{c,k_{c}}}\\ \vdots \\ {a^{*}}_{c,k_{c}}{\upsilon^{*}}_{N_{c,k_{c}}} \end{array} \end{array}\right]\;\\ \;={\left[\begin{array}{c} {\upsilon^{*}}_{1_{c,k_{c}}}\\ \begin{array}{c} {\upsilon^{*}}_{2_{c,k_{c}}}\\ \vdots \\ {\upsilon^{*}}_{N_{c,k_{c}}} \end{array} \end{array}\right]}^{H}blkdiag{\left[{a^{*}}_{c,k_{c}},\;{a^{*}}_{c,k_{c}},\;\cdots ,\;{a^{*}}_{c,k_{c}}\right]}^{H}.\;\\ E_{n2}E_{n2}^{H}blkdiag\left[{a^{*}}_{c,k_{c}},\;{a^{*}}_{c,k_{c}},\;\cdots ,\;{a^{*}}_{c,k_{c}}\right]\left[\begin{array}{c} {\upsilon^{*}}_{1_{c,k_{c}}}\\ \begin{array}{c} {\upsilon^{*}}_{2_{c,k_{c}}}\\ \vdots \\ {\upsilon^{*}}_{N_{c,k_{c}}} \end{array} \end{array}\right]=0 \end{array}$$

As proven in [27], Equations (37) and (38) are equivalent to each other; therefore, the estimator over θ , which corresponds to circular signals, can obtained based on Equation (37). Since:

(39) $$\left[\begin{array}{c} \upsilon_{1_{c,k_{c}}}\\ \begin{array}{c} \upsilon_{2_{c,k_{c}}}\\ \vdots \\ \upsilon_{N_{c,k_{c}}} \end{array} \end{array}\right]\neq 0$$

Defining a vector $p_{c}\left(\tilde{l}\right)={\left[1,\tilde{l},...,{\tilde{l}}^{M-1}\right]}^{T}$ only related to $\tilde{l}$ , where l˜=e−j(2π(2πλλ)dxcosθc,kc . Therefore, we can get formula $p_{c}\left(\tilde{l}\right)=a_{c,k_{c}}$ , then defining a NM×N matrix Λ ( $\tilde{l}$ ) only related to $\tilde{l}$ ,

(40) $$\begin{array}{c} \Lambda \left(\tilde{l}\right)=blkdiag\left[p_{c}\left(\tilde{l}\right),p_{c}\left(\tilde{l}\right),\cdots ,p_{c}\left(\tilde{l}\right)\right]=blkdiag\left[a_{c,k_{c}},a_{c,k_{c}},\cdots ,a_{c,k_{c}}\right] \end{array}$$

and:

(41) $$Q_{c}\left(\tilde{l}\right)={\tilde{l}}^{M-1}\Lambda^{T}\left({\tilde{l}}^{-1}\right)E_{n1}E_{n1}^{H}\Lambda \left(\tilde{l}\right)$$

Note that if $(2NM-K_{n}-2K_{c})\geq N$ and when θ is not the true angle of circular signal, $Q_{c}\left(\tilde{l}\right)$ is of full rank because in this case, the column rank of $E_{n1}$ is not less than *N*. Then, Equation (37) holds true only when θ equals the true angle of circular signal ( $Q_{c}\left(\tilde{l}\right)$ drops rank). Since the covariance matrix of $\overset{⌣}{\;f}$ is obtained from a finite number of samples, the reduction of the rank of $Q_{c}\left(\tilde{l}\right)$ can roughly be replaced by the minimum of the determinant of $Q_{c}\left(\tilde{l}\right)$ . Therefore, we get the estimator of θ about circular signals as follows:

(42) $$f_{c}\left(\tilde{l}\right)=\det\left(Q_{c}\left(\tilde{l}\right)\right)=\det\left({\tilde{l}}^{M-1}\Lambda^{T}\left({\tilde{l}}^{-1}\right)E_{n1}E_{n1}^{H}\Lambda \left(\tilde{l}\right)\right)$$

Notice that $f_{c}\left(\tilde{l}\right)$ is a 2×N(M−1) order polynomial, which means that there are N(M−1) pairs of conjugated roots, and the estimated $K_{c}$ signal DOAs of θ can be obtained by finding the closest roots of the unit circle, which are given by:

(43) $${\hat{\theta }}_{k_{c}}=\arccos\left[-\frac{\lambda }{2\pi d_{x}}\arg\left({\tilde{l}}_{k_{c}}\right)\right],k_{c}=1,...,K_{c}$$

where ${\tilde{l}}_{k_{c}},k_{c}=1,...,K_{c}$ are the roots closest to the unit circle. Then, we take the estimated ${\hat{\theta }}_{k_{c}}$ of circular signals into Equation (37) to get the estimator of β :

(44) $$f_{c}^{\prime }\left(\tilde{u}\right)=\det\left(Q_{c}^{\prime }\left(\tilde{u}\right)\right)$$

where:

(45) $$Q_{c}^{\prime }\left(\tilde{u}\right)={\tilde{u}}^{\left(N-1\right)}\Psi^{T}\left({\tilde{u}}^{-1}\right){\tilde{l}}^{M-1}\Lambda^{T}\left({\tilde{l}}^{-1}\right)E_{n1}E_{n1}^{H}\Lambda \left(\tilde{l}\right)\Psi \left(\tilde{u}\right)$$

u˜=ej(2π(2πλλ)dycosβc,kc ; this means that $\upsilon_{m_{c,k_{c}}}={\tilde{u}}^{m-1},\;m=1,\;\cdots \;N$ and:

(46) $$\Psi \left(\tilde{u}\right)=\left[\begin{array}{c} 1\\ \begin{array}{c} \tilde{u}\\ \vdots \\ {\tilde{u}}^{N-1} \end{array} \end{array}\right]=\left[\begin{array}{c} \upsilon_{1_{c,k_{c}}}\left(\beta \right)\\ \begin{array}{c} \upsilon_{2_{c,k_{c}}}\left(\beta \right)\\ \vdots \\ \upsilon_{N_{c,k_{c}}}\left(\beta \right) \end{array} \end{array}\right]$$

To obtain the estimate of β , we need to solve the roots of $f_{c}^{\prime }\left(\tilde{u}\right)$ . According to $f_{c}^{\prime }\left(\tilde{u}\right)$ being a 2×(N−1) -order polynomial, this means there are N−1 pairs of conjugated roots. The estimate of β can be obtained by finding the closest roots of the unit circle. Therefore, we can automatically pair the closest roots ${\tilde{u}}_{1},\;{\tilde{u}}_{2},\;...,\;{\tilde{u}}_{K_{c}}$ corresponding to ${\hat{\theta }}_{1},\;{\hat{\theta }}_{2},\;\ldots ,\;{\hat{\theta }}_{K_{c}}$ , respectively, and the DOAs of β are given by:

(47) $${\hat{\beta }}_{k_{c}}=\arccos\left[\frac{\lambda }{2\pi d_{y}}\arg\left({\tilde{u}}_{k_{c}}\right)\right],k_{c}=1,\;...,\;K_{c}$$

where ${\tilde{u}}_{k_{c}},k_{c}=1,\;...,\;K_{c}$ are the roots closest to the unit circle, respectively.

### 3.3. Identification of Circular and Noncircular Signals

In order to distinguish the 2D DOAs of circular and noncircular signals from the mixed signals, Equation (34) can be changed into:

(48) $$\begin{array}{cc} E_{n}^{H}{\overset{⌣}{\;c}}_{c,k_{c}} & =E_{n}^{H}\left[\begin{array}{cc} a_{c,k_{c}}\upsilon_{1_{{}_{c,k_{c}}}} & 0_{M\times 1}\\ a_{c,k_{c}}\upsilon_{2_{c,k_{c}}} & 0_{M\times 1}\\ \vdots & \vdots \\ a_{c,k_{c}}\upsilon_{N_{{}_{c,k_{c}}}} & 0_{M\times 1}\\ 0_{M\times 1} & a_{c,k_{c}}^{*}{\upsilon^{*}}_{1_{{}_{c,k_{c}}}}\\ 0_{M\times 1} & a_{c,k_{c}}^{*}{\upsilon^{*}}_{2_{{}_{c,k_{c}}}}\\ \vdots & \vdots \\ 0_{M\times 1} & a_{c,k_{c}}^{*}{\upsilon^{*}}_{N_{{}_{c,k_{c}}}} \end{array}\right]\\ & =\;E_{n}^{H}\;blkdiag\left[a_{c,k_{c}},\;a_{c,k_{c}},\;\cdots ,\;a_{c,k_{c}},\;a_{c,k_{c}}^{*},\;a_{c,k_{c}}^{*},\;\cdots ,a_{c,k_{c}}^{*}\right].\\ & \;\left[\begin{array}{cc} \upsilon_{1_{{}_{c,k_{c}}}} & 0\\ \begin{array}{c} \upsilon_{2_{{}_{c,k_{c}}}}\\ \vdots \\ \upsilon_{N_{{}_{c,k_{c}}}} \end{array} & \begin{array}{c} 0\\ \vdots \\ 0 \end{array}\\ 0 & \upsilon_{1_{c,k_{c}}}^{*}\\ \begin{array}{c} 0\\ \vdots \\ 0 \end{array} & \begin{array}{c} \upsilon_{2_{c,k_{c}}}^{*}\\ \vdots \\ \upsilon_{N_{c,k_{c}}}^{*} \end{array} \end{array}\right]=0 \end{array}$$

due to $\left[\begin{array}{cc} \upsilon_{1_{c,k_{c}}} & 0\\ \begin{array}{c} \upsilon_{2_{c,k_{c}}}\\ \vdots \\ \upsilon_{N_{c,k_{c}}} \end{array} & \begin{array}{c} 0\\ \vdots \\ 0 \end{array}\\ 0 & \upsilon_{1_{c,k_{c}}}^{*}\\ \begin{array}{c} 0\\ \vdots \\ 0 \end{array} & \begin{array}{c} \upsilon_{2_{c,k_{c}}}^{*}\\ \vdots \\ \upsilon_{N_{c,k_{c}}}^{*} \end{array} \end{array}\right]\neq 0$ , we can utilize method of noncircular to establish the estimator over θ and β of circular signals as follows:

(49) $$\det({\tilde{l}}^{M-1}\Omega^{T}\left({\tilde{l}}^{-1}\right)E_{n}E_{n}^{H}{\tilde{l}}^{M-1}\Omega \left(\tilde{l}\right))=0$$

and:

(50) $$\det({\tilde{u}}^{\left(N-1\right)}\Theta^{T}\left({\tilde{u}}^{-1}\right)\Omega^{T}\left({\tilde{l}}^{-1}\right)E_{n}E_{n}^{H}\Omega \left(\tilde{l}\right){\tilde{u}}^{\left(N-1\right)}\Theta \left(\tilde{u}\right))=0$$

This means the noncircular method can also be applied to solve circular signals. Therefore, we can achieve the 2D DOAs of both non-circular and circular sources from Equations (28)–(30) and (33) and only obtain the 2D DOAs of circular sources from Equations (42)–(44) and (47). Then, the purpose of distinguishing the circular and noncircular signals from the mixtures can be accomplished.

The proposed algorithm can be outlined as:

Step 1: Construct the new data vector $\overset{⌣}{\;f}$ and calculate its approximate covariance $\hat{\overset{⌣}{\;R}}$ from Equations (14) and (22).
Step 2: Perform EVDto $\hat{\overset{⌣}{\;R}}$ and achieve the noise subspace ${\hat{E}}_{n}$ from Equation (23).
Step 3: Estimate the *K* 2D DOAs of the mixed signals from Equations (28)–(30) and (33).
Step 4: Obtain ${\hat{E}}_{n1}$ by partitioning the matrix ${\hat{E}}_{n}$ .
Step 5: Estimate the $K_{c}$ 2D DOAs of the circular signals from Equations (42)–(44) and (47).
Step 6: Compare the estimate achieved by Step 3 and Step 5 to distinguish the $K_{n}$ 2D DOAs of the noncircular signals and the $K_{c}$ 2D DOAs of the circular ones.

**Remark** **2.** *To calculate $\hat{\overset{⌣}{\;R}}$ , a computational complexity of $O({\left(2NM\right)}^{2}L)$ is needed. The computational complexity of eigendecomposition operation is $O({\left(2NM\right)}^{3})$ . The proposed method employs four 1D estimators of polynomial-rooting; therefore, the complexity for the proposed method is $O\left({\left(2NM\right)}^{2}L+{\left(2NM\right)}^{3}+4NM\times K+2NM\times K_{c}\right)$ . While the algorithm in [27] employs several 1D spatial spectrum search procedures to obtain the 2D DOAs of signals, by defining the scanning interval of θ∈[0,π] with an interval of Δθ and β∈[0,π] with an interval of Δβ , respectively, the complexity for the algorithm in [27] is $O({\left(2NM\right)}^{2}L+{\left(2NM\right)}^{3}+\frac{\pi }{\Delta \theta }{\left(2NM\right)}^{2}+K\frac{\pi }{\Delta \beta }{\left(2NM\right)}^{2}+\frac{\pi }{\Delta \theta }{\left(NM\right)}^{2}+K_{c}\frac{\pi }{\Delta \beta }{\left(NM\right)}^{2})$ . The algorithm in [16] employs two direct 2D spatial spectrum search procedures, whose complexity for the method is $O\left({\left(NM\right)}^{2}L+{\left(NM\right)}^{3}+\frac{\pi }{\Delta \theta }\frac{\pi }{\Delta \beta }{\left(2NM\right)}^{2}+\frac{\pi }{\Delta \theta }\frac{\pi }{\Delta \beta }{\left(NM\right)}^{2}\right)$ . Therefore, the computational complexity of the proposed method has been reduced greatly*.

## 4. Theoretical Performance Analysis

The theoretical DOA estimation error is caused by the finite data effect, the sensor errors and the unknown noise structure [31,35], and in this section, we will study the theoretical perturbation of the proposed algorithm as a criterion for evaluation.

### 4.1. Theoretical Perturbation for Non-Circular Sources

According to Equation (25), defining $a_{n,k_{n}}={\left[1,\;l,\;...,\;l^{M-1}\right]}^{T}$ , $\upsilon_{m_{n,k_{n}}}=u^{m-1},\;m=1,\;\cdots ,\;N$ and $b_{n,k_{n}}=z$ , where l=e−j(2π(2πλλ)dxcosθn,kn , u=ej(2π(2πλλ)dycosβn,kn , $z=e^{j\varphi_{n,k_{n}}}$ , $\varphi_{n,k_{n}}$ is the non-circular phase of non-circular sources. Therefore, Equation (25) can be changed into:

(51) $$\begin{array}{c} E_{n}^{H}\;blkdiag\left[a_{n,k_{n}},\;a_{n,k_{n}},\;\cdots ,\;a_{n,k_{n}},\;a_{n,k_{n}}^{*},\;a_{n,k_{n}}^{*},\;\cdots ,\;a_{n,k_{n}}^{*}\right]\left[\begin{array}{cc} 1 & 0\\ \begin{array}{c} u\\ \vdots \\ u^{N-1} \end{array} & \begin{array}{c} 0\\ \vdots \\ 0 \end{array}\\ 0 & 1\\ \begin{array}{c} 0\\ \vdots \\ 0 \end{array} & \begin{array}{c} u^{*}\\ \vdots \\ {u^{*}}^{N-1} \end{array} \end{array}\right]\left[\begin{array}{c} z\\ z^{-1} \end{array}\right]=0 \end{array}$$

Defining:

(52) $$\begin{array}{cc} M\left(l,u,z\right)= & \;blkdiag\left[a_{n,k_{n}},\;a_{n,k_{n}},\;\cdots ,\;a_{n,k_{n}},\;a_{n,k_{n}}^{*},\;a_{n,k_{n}}^{*},\;\cdots ,\;a_{n,k_{n}}^{*}\right].\;\\ & \left[\begin{array}{cc} 1 & 0\\ \begin{array}{c} u\\ \vdots \\ u^{N-1} \end{array} & \begin{array}{c} 0\\ \vdots \\ 0 \end{array}\\ 0 & 1\\ \begin{array}{c} 0\\ \vdots \\ 0 \end{array} & \begin{array}{c} u^{*}\\ \vdots \\ {u^{*}}^{N-1} \end{array} \end{array}\right]\left[\begin{array}{c} z\\ z^{-1} \end{array}\right] \end{array}$$

Then, we can define the equation as follows:

(53) $$f\left(l,u,z\right)=M^{H}\left(l,u,z\right)E_{n}E_{n}^{H}M\left(l,u,z\right)$$

In practical situations, due to the influence of noise, the sensor errors and the finite data effect, we can only achieve ${\hat{E}}_{n}$ , the approximation of $E_{n}$ , according to Equation (53), we can get the following formula:

(54) $$\hat{f}\left(l,u,z\right)=M^{H}\left(l,u,z\right){\hat{E}}_{n}{\hat{E}}_{n}^{H}M\left(l,u,z\right)$$

The locations of the local minima of either the noise-free or perturbed spectral polynomials are obtained when the first derivative equal to zero. In other words, the roots of the derivative of $\hat{f}\left(l,\;u,\;z\right)$ give the locations of the relative minima of $\hat{f}\left(l,\;u,\;z\right)$ . Thus, to find the perturbations of the DOAs (which correspond to minima of $\hat{f}\left(l,\;u,\;z\right)$ ), we must calculate the perturbations of the roots of the derivative of $\hat{f}\left(l,\;u,\;z\right)$ . Define $\left({\hat{l}}_{k_{n}},\;{\hat{u}}_{k_{n}},\;{\hat{z}}_{k_{n}}\right)$ as the estimate of (l,u,z) when the true angle is $\theta =\theta_{n,k_{n}}$ , $\beta =\beta_{n,k_{n}}$ , and $\varphi =\varphi_{n,k_{n}}$ . Therefore, the first-order partial derivatives of $\hat{f}\left(l,u,z\right)$ at $\left({\hat{l}}_{k_{n}},\;{\hat{u}}_{k_{n}},\;{\hat{z}}_{k_{n}}\right)$ are zeros. Approximating the perturbation of DOAs uses the first two terms in the Taylor series expansion of the first-order partial derivative of $\hat{f}\left(l,u,z\right)$ about the true angles of arrival.

(55) $${\hat{f}}_{l}^{\prime }\left(k_{n}\right)+\Delta l_{k_{n}}{\hat{f}}_{l,l}^{\prime \prime }\left(k_{n}\right)+\Delta u_{k_{n}}{\hat{f}}_{l,u}^{\prime \prime }\left(k_{n}\right)+\Delta z_{k_{n}}{\hat{f}}_{l,z}^{\prime \prime }\left(k_{n}\right)=0$$

(56) $${\hat{f}}_{u}^{\prime }\left(k_{n}\right)+\Delta l_{k_{n}}{\hat{f}}_{u,l}^{\prime \prime }\left(k_{n}\right)+\Delta u_{k_{n}}{\hat{f}}_{u,u}^{\prime \prime }\left(k_{n}\right)+\Delta z_{k_{n}}{\hat{f}}_{u,z}^{\prime \prime }\left(k_{n}\right)=0$$

(57) $${\hat{f}}_{z}^{\prime }\left(k_{n}\right)+\Delta l_{k_{n}}{\hat{f}}_{z,l}^{\prime \prime }\left(k_{n}\right)+\Delta u_{k_{n}}{\hat{f}}_{z,u}^{\prime \prime }\left(k_{n}\right)+\Delta z_{k_{n}}{\hat{f}}_{z,z}^{\prime \prime }\left(k_{n}\right)=0$$

where $\Delta l_{k_{n}}={\hat{l}}_{k_{n}}-l_{k_{n}}$ , $\Delta u_{k_{n}}={\hat{u}}_{k_{n}}-u_{k_{n}}$ and $\Delta z_{k_{n}}={\hat{z}}_{k_{n}}-z_{k_{n}}$ are the perturbations of $l_{k_{n}}$ , $u_{k_{n}}$ and $z_{k_{n}}$ , respectively. Together with Equations (55)–(57), solve the set of equations as follows:

(58) $$\begin{array}{cc} \Delta l_{k_{n}} & =\frac{1}{\delta }\left|\begin{array}{ccc} -{{\hat{f}}^{\prime }}_{l}\left(k_{n}\right) & {{\hat{f}}^{\prime \prime }}_{l,u}\left(k_{n}\right) & {{\hat{f}}^{\prime \prime }}_{l,z}\left(k_{n}\right)\\ -{{\hat{f}}^{\prime }}_{u}\left(k_{n}\right) & {{\hat{f}}^{\prime \prime }}_{u,u}\left(k_{n}\right) & {{\hat{f}}^{\prime \prime }}_{u,z}\left(k_{n}\right)\\ -{{\hat{f}}^{\prime }}_{z}\left(k_{n}\right) & {{\hat{f}}^{\prime \prime }}_{z,u}\left(k_{n}\right) & {{\hat{f}}^{\prime \prime }}_{z,z}\left(k_{n}\right) \end{array}\right|\\ & =\frac{1}{\delta }\cdot \{{{\hat{f}}^{\prime }}_{l}\left(k_{n}\right)\left[{\left({{\hat{f}}^{\prime \prime }}_{u,z}\left(k_{n}\right)\right)}^{2}-{{\hat{f}}^{\prime \prime }}_{u,u}\left(k_{n}\right){{\hat{f}}^{\prime \prime }}_{z,z}\left(k_{n}\right)\right]+{{\hat{f}}^{\prime }}_{u}\left(k_{n}\right)\left[{{\hat{f}}^{\prime \prime }}_{l,u}\left(k_{n}\right){{\hat{f}}^{\prime \prime }}_{z,z}\left(k_{n}\right)-{{\hat{f}}^{\prime \prime }}_{l,z}\left(k_{n}\right){{\hat{f}}^{\prime \prime }}_{u,z}\left(k_{n}\right)\right]\\ & \;+{{\hat{f}}^{\prime }}_{z}\left(k_{n}\right)\left[{{\hat{f}}^{\prime \prime }}_{l,z}\left(k_{n}\right){{\hat{f}}^{\prime \prime }}_{u,u}\left(k_{n}\right)-{{\hat{f}}^{\prime \prime }}_{l,u}\left(k_{n}\right){{\hat{f}}^{\prime \prime }}_{u,z}\left(k_{n}\right)\right]\} \end{array}$$

(59) $$\begin{array}{cc} \Delta u_{k_{n}} & =\frac{1}{\delta }\left|\begin{array}{ccc} {{\hat{f}}^{\prime \prime }}_{l,l}\left(k_{n}\right) & -{{\hat{f}}^{\prime }}_{l}\left(k_{n}\right) & {{\hat{f}}^{\prime \prime }}_{l,z}\left(k_{n}\right)\\ {{\hat{f}}^{\prime \prime }}_{u,l}\left(k_{n}\right) & -{{\hat{f}}^{\prime }}_{u}\left(k_{n}\right) & {{\hat{f}}^{\prime \prime }}_{u,z}\left(k_{n}\right)\\ {{\hat{f}}^{\prime \prime }}_{z,l}\left(k_{n}\right) & -{{\hat{f}}^{\prime }}_{z}\left(k_{n}\right) & {{\hat{f}}^{\prime \prime }}_{z,z}\left(k_{n}\right) \end{array}\right|\\ & =\frac{1}{\delta }\cdot \{{{\hat{f}}^{\prime }}_{l}\left(k_{n}\right)\left[{{\hat{f}}^{\prime \prime }}_{u,l}\left(k_{n}\right){{\hat{f}}^{\prime \prime }}_{z,z}\left(k_{n}\right)-{{\hat{f}}^{\prime \prime }}_{u,z}\left(k_{n}\right){{\hat{f}}^{\prime \prime }}_{z,l}\left(k_{n}\right)\right]+{{\hat{f}}^{\prime }}_{u}\left(k_{n}\right)\left[{\left({{\hat{f}}^{\prime \prime }}_{l,z}\left(k_{n}\right)\right)}^{2}-{{\hat{f}}^{\prime \prime }}_{l,l}\left(k_{n}\right){{\hat{f}}^{\prime \prime }}_{z,z}\left(k_{n}\right)\right]\\ & \;+{{\hat{f}}^{\prime }}_{z}\left(k_{n}\right)\left[{{\hat{f}}^{\prime \prime }}_{l,l}\left(k_{n}\right){{\hat{f}}^{\prime \prime }}_{u,z}\left(k_{n}\right)-{{\hat{f}}^{\prime \prime }}_{l,z}\left(k_{n}\right){{\hat{f}}^{\prime \prime }}_{u,l}\left(k_{n}\right)\right]\} \end{array}$$

where:

(60) $$\delta =\left|\begin{array}{ccc} {{\hat{f}}^{\prime \prime }}_{l,l}\left(k_{n}\right) & {{\hat{f}}^{\prime \prime }}_{l,u}\left(k_{n}\right) & {{\hat{f}}^{\prime \prime }}_{l,z}\left(k_{n}\right)\\ {{\hat{f}}^{\prime \prime }}_{u,l}\left(k_{n}\right) & {{\hat{f}}^{\prime \prime }}_{u,u}\left(k_{n}\right) & {{\hat{f}}^{\prime \prime }}_{u,z}\left(k_{n}\right)\\ {{\hat{f}}^{\prime \prime }}_{z,l}\left(k_{n}\right) & {{\hat{f}}^{\prime \prime }}_{z,u}\left(k_{n}\right) & {{\hat{f}}^{\prime \prime }}_{z,z}\left(k_{n}\right) \end{array}\right|$$

According to the expressions for Equation (54), the first derivative, ${\hat{f}}_{\eta }^{\prime }\left(k_{n}\right)\left(\eta =l,\;u,\;z\right)$ , and the second derivative, ${\hat{f}}_{\tau ,\kappa }^{\prime \prime }\left(k_{n}\right)\left(\tau ,\kappa =l,\;u,\;z\right)$ , can be achieved, respectively. Using the value of $\left(l_{k_{n}},\;u_{k_{n}},\;z_{k_{n}}\right)$ and Equations (58) and (59), we can obtain the perturbation Δl and Δu . Then, the perturbation of θ and β can be achieved from Δl and Δu , respectively. The relation between an arrival angle and a signal root is given in [36]:

(61) Δθn,kn=Cθ,knImΔlknlkn

(62) Δβn,kn=Cβ,knImΔuknukn

where $C_{\theta ,k_{n}}=\frac{\lambda }{2\pi d_{x}\sin\theta_{n,k_{n}}}$ and $C_{\beta ,k_{n}}=-\frac{\lambda }{2\pi d_{y}\sin\beta_{n,k_{n}}}$ . Therefore, the theoretical perturbation of $\theta_{n,k_{n}}$ and $\beta_{n,k_{n}}$ can be obtained from Equations (61) and (62).

### 4.2. Theoretical Perturbation for Circular Sources

Similar to the noncircular method, defining $a_{c,k_{c}}={\left[1,\tilde{l},...,{\tilde{l}}^{M-1}\right]}^{T}$ and $\upsilon_{m_{c,k_{c}}}={\tilde{u}}^{m-1},\;m=1,\cdots N$ , where l˜=e−j(2π(2πλλ)dxcosθc,kc and u˜=ej(2π(2πλλ)dycosβc,kc . Equation (35) can be changed to:

(63) $$\;E_{n1}^{H}\left[\begin{array}{cccc} a_{c,k_{c}} & & & \\ & a_{c,k_{c}} & & \\ & & \ddots & \\ & & & a_{c,k_{c}} \end{array}\right]\left[\begin{array}{c} 1\\ \begin{array}{c} \tilde{u}\\ \vdots \\ {\tilde{u}}^{N-1} \end{array} \end{array}\right]=0$$

defining:

(64) $$\;M\left(\tilde{l},\tilde{u}\right)=\left[\begin{array}{cccc} a_{c,k_{c}} & & & \\ & a_{c,k_{c}} & & \\ & & \ddots & \\ & & & a_{c,k_{c}} \end{array}\right]\left[\begin{array}{c} 1\\ \begin{array}{c} \tilde{u}\\ \vdots \\ {\tilde{u}}^{N-1} \end{array} \end{array}\right]$$

therefore, the equation can be defined as follows:

(65) $$f\left(\tilde{l},\tilde{u}\right)=M^{H}\left(\tilde{l},\tilde{u}\right)E_{n1}E_{n1}^{H}M\left(\tilde{l},\tilde{u}\right)$$

In practical application, we can only achieve ${\hat{E}}_{n1}$ , the approximation of $E_{n1}$ ; according to Equation (65), we can get the following equation:

(66) $$\hat{f}\left(\tilde{l},\tilde{u}\right)=M^{H}\left(\tilde{l},\tilde{u}\right){\hat{E}}_{n1}{\hat{E}}_{n1}^{H}M\left(\tilde{l},\tilde{u}\right)$$

the roots of the derivative of $\hat{f}\left(\tilde{l},\tilde{u}\right)$ give the locations of the relative minima of $\hat{f}\left(\tilde{l},\tilde{u}\right)$ . Define $\left({\hat{\tilde{l}}}_{k_{c}},{\hat{\tilde{u}}}_{k_{c}}\right)$ as the estimate of $\left(\tilde{l},\tilde{u}\right)$ when the true angle is $\theta =\theta_{c,k_{c}}$ and $\beta =\beta_{c,k_{c}}$ . Therefore, the first-order partial derivatives of $\hat{f}\left(\tilde{l},\tilde{u}\right)$ at $\left({\hat{\tilde{l}}}_{k_{c}},{\hat{\tilde{u}}}_{k_{c}}\right)$ are zeros. Approximating the perturbation of DOAs uses the first two terms in the Taylor series expansion of the first-order partial derivative of $\hat{f}\left(\tilde{l},\tilde{u}\right)$ at the true angles of arrival.

(67) $${\hat{f}}_{\tilde{l}}^{\prime }\left(k_{c}\right)+\Delta {\tilde{l}}_{k_{c}}{\hat{f}}_{\tilde{l},\tilde{l}}^{\prime \prime }\left(k_{c}\right)+\Delta {\tilde{u}}_{k_{c}}{\hat{f}}_{\tilde{l},\tilde{u}}^{\prime \prime }\left(k_{c}\right)=0$$

(68) $${\hat{f}}_{\tilde{u}}^{\prime }\left(k_{c}\right)+\Delta {\tilde{l}}_{k_{c}}{\hat{f}}_{\tilde{u},\tilde{l}}^{\prime \prime }\left(k_{c}\right)+\Delta {\tilde{u}}_{k_{c}}{\hat{f}}_{\tilde{u},\tilde{u}}^{\prime \prime }\left(k_{c}\right)=0$$

where $\Delta {\tilde{l}}_{k_{c}}={\hat{\tilde{l}}}_{k_{c}}-{\tilde{l}}_{k_{c}}$ and $\Delta {\tilde{u}}_{k_{c}}={\hat{\tilde{u}}}_{k_{c}}-{\tilde{u}}_{k_{c}}$ are the perturbations of ${\tilde{l}}_{k_{c}}$ and ${\tilde{u}}_{k_{c}}$ , respectively. Together with Equations (67) and (68) to solve the set of equations as follows:

(69) $$\begin{array}{c} \Delta {\tilde{l}}_{k_{c}}=\frac{1}{\tilde{\delta }}\left|\begin{array}{cc} -{{\hat{f}}^{\prime }}_{\tilde{l}}\left(k_{c}\right) & {{\hat{f}}^{\prime \prime }}_{\tilde{l},\tilde{u}}\left(k_{c}\right)\\ -{{\hat{f}}^{\prime }}_{\tilde{u}}\left(k_{c}\right) & {{\hat{f}}^{\prime \prime }}_{\tilde{u},\tilde{u}}\left(k_{c}\right) \end{array}\right|=\frac{1}{\tilde{\delta }}\cdot \left[{{\hat{f}}^{\prime }}_{\tilde{u}}\left(k_{c}\right){{\hat{f}}^{\prime \prime }}_{\tilde{l},\tilde{u}}\left(k_{c}\right)-{{\hat{f}}^{\prime }}_{\tilde{l}}\left(k_{c}\right){{\hat{f}}^{\prime \prime }}_{\tilde{u},\tilde{u}}\left(k_{c}\right)\right] \end{array}$$

(70) $$\begin{array}{c} \Delta {\tilde{u}}_{k_{c}}=\frac{1}{\tilde{\delta }}\left|\begin{array}{cc} {{\hat{f}}^{\prime \prime }}_{\tilde{l},\tilde{l}}\left(k_{c}\right) & -{{\hat{f}}^{\prime }}_{\tilde{l}}\left(k_{c}\right)\\ {{\hat{f}}^{\prime \prime }}_{\tilde{u},\tilde{l}}\left(k_{c}\right) & -{{\hat{f}}^{\prime }}_{\tilde{u}}\left(k_{c}\right) \end{array}\right|=\frac{1}{\tilde{\delta }}\cdot \left[{{\hat{f}}^{\prime }}_{\tilde{l}}\left(k_{c}\right){{\hat{f}}^{\prime \prime }}_{\tilde{u},\tilde{l}}\left(k_{c}\right)-{{\hat{f}}^{\prime }}_{\tilde{u}}\left(k_{c}\right){{\hat{f}}^{\prime \prime }}_{\tilde{l},\tilde{l}}\left(k_{c}\right)\right] \end{array}$$

where:

(71) $$\tilde{\delta }=\left|\begin{array}{cc} {{\hat{f}}^{\prime \prime }}_{\tilde{l},\tilde{l}}\left(k_{c}\right) & {{\hat{f}}^{\prime \prime }}_{\tilde{l},\tilde{u}}\left(k_{c}\right)\\ {{\hat{f}}^{\prime \prime }}_{\tilde{u},\tilde{l}}\left(k_{c}\right) & {{\hat{f}}^{\prime \prime }}_{\tilde{u},\tilde{u}}\left(k_{c}\right) \end{array}\right|$$

According to the expressions for Equation (66), the first derivative, ${\hat{f}}_{\tilde{\eta }}^{\prime }\left(k_{c}\right)\left(\tilde{\eta }=\tilde{l},\tilde{u}\right)$ and the second derivative, ${\hat{f}}_{\tilde{\tau },\tilde{\kappa }}^{\prime \prime }\left(k_{c}\right),\left(\tilde{\tau },\tilde{\kappa }=\tilde{l},\tilde{u}\right)$ can be achieved, respectively. Using the value of $\left({\tilde{l}}_{k_{c}},{\tilde{u}}_{k_{c}}\right)$ and Equations (69) and (70), we can obtain the perturbation $\Delta \tilde{l}$ and $\Delta \tilde{u}$ . Then, the relation between an arrival angle and a signal root is as follows:

(72) Δθc,kc=Cθ,kcImΔl˜kcl˜kc

(73) Δβc,kc=Cβ,kcImΔu˜kcu˜kc

where $C_{\theta ,k_{c}}=\frac{\lambda }{2\pi d_{x}\sin\theta_{c,k_{c}}}$ and $C_{\beta ,k_{c}}=-\frac{\lambda }{2\pi d_{y}\sin\beta_{c,k_{c}}}$ ; therefore, the theoretical perturbation of $\theta_{c,k_{c}}$ and $\beta_{c,k_{c}}$ can be obtained.

## 5. Simulation Results

In this section, simulation results are provided to demonstrate the performance of the proposed algorithm. For Simulations (1)–(3), the URAs have N=3 rows and M=8 columns; both $d_{x}$ and $d_{y}$ are half wavelength. For all simulations, we utilize BPSK and QPSK to represent the strictly non-circular signal and the circular signal, respectively, and the sources can be realized base on (7). For instance, if we take $\eta_{k}=0$ and $\varphi_{k}=0$ , the QPSK signal $s_{k}\in \left(\frac{\sqrt{2}}{2}+\frac{\sqrt{2}}{2}j,\frac{\sqrt{2}}{2}-\frac{\sqrt{2}}{2}j,-\frac{\sqrt{2}}{2}+\frac{\sqrt{2}}{2}j,-\frac{\sqrt{2}}{2}-\frac{\sqrt{2}}{2}j\right)$ can be obtained. If we take $\eta_{k}=1$ and $\varphi_{k}=0$ , the BPSK signal $s_{k}\in \left(-1,+1\right)$ can be acquired. The power of additive white Gaussian noise is $\sigma_{n}^{2}$ , and the signal-to-noise (*SNR*) is defined as $SNR=10\log_{10}\left(\sigma_{s}^{2}/\sigma_{n}^{2}\right)$ . We use the root mean square error (RMSE) to evaluate the estimation performance, which is defined as:

(74) $$RMSE=\sqrt{\frac{1}{KM_{c}}\sum_{k=1}^{K}\sum_{m=1}^{M_{c}}\left[{\left({\hat{\varepsilon }}_{m,k}-\varepsilon_{k}\right)}^{2}\right]}$$

where $M_{c}$ is the number of Monte Carlo simulations, *K* is the number of signals, ${\hat{\varepsilon }}_{m,k}$ is the estimated $\theta_{k}$ or $\beta_{k}$ in the *m*-th Monte Carlo simulation and $\varepsilon_{k}$ is the true value for either $\theta_{k}$ or $\beta_{k}$ of the *k*-th signal.

### 5.1. The 2D DOAs’ Scattergram of the Estimators

**Figure 2:**
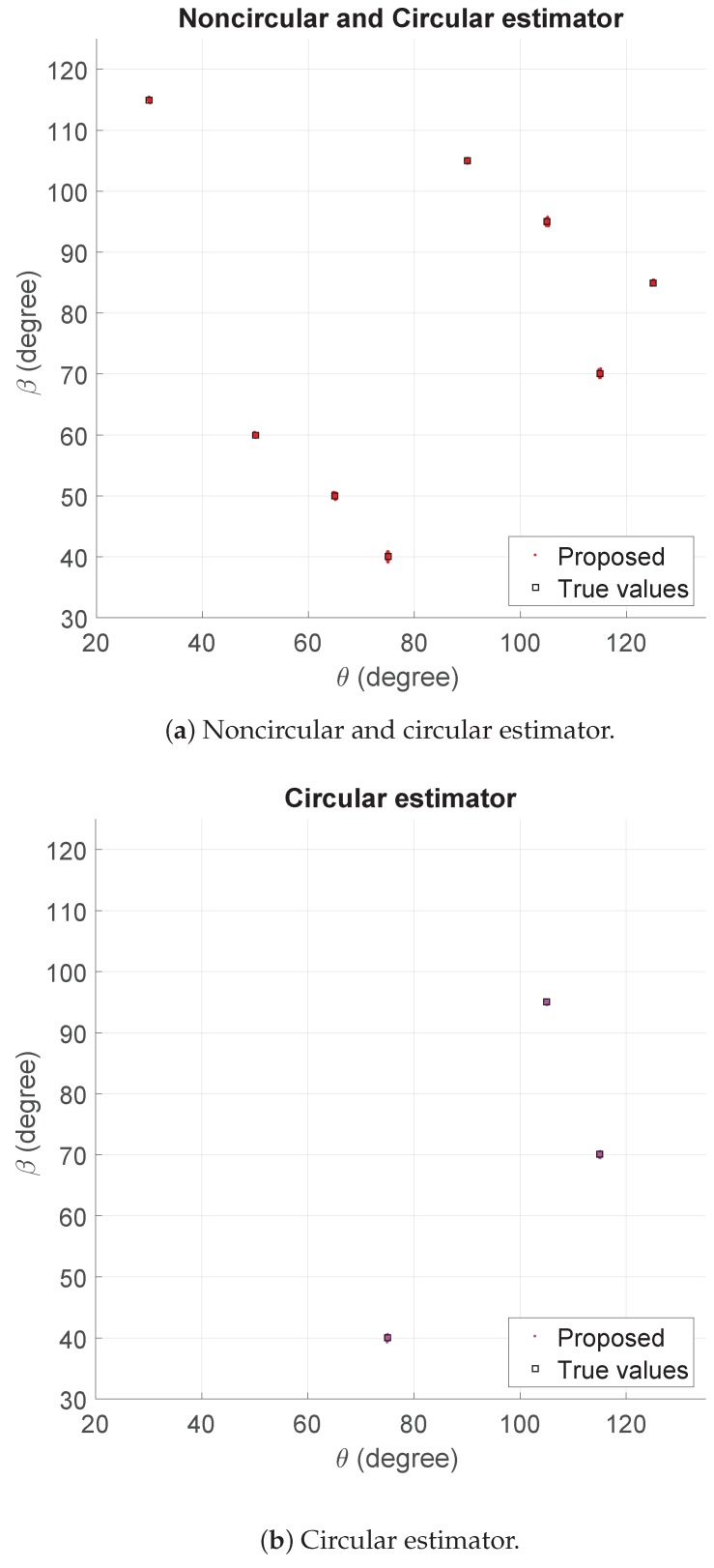
The estimators for noncircular and circular signals using the proposed algorithm when SNR = 10 dB and the number of snapshots = 500.

To demonstrate the performance of the proposed algorithm, we examine the scattergram of 2D θ and β of the method. Five BPSK signals and three QPSK signals are considered here. The BPSK signals are from the directions $\left(\theta_{1}=65^{\circ },\beta_{1}=50^{\circ }\right)$ , $\left(\theta_{2}=90^{\circ },\beta_{2}=105^{\circ }\right)$ , $\left(\theta_{3}=50^{\circ },\beta_{3}=60^{\circ }\right)$ , $\left(\theta_{4}=125^{\circ },\beta_{4}=85^{\circ }\right)$ and $\left(\theta_{5}=30^{\circ },\beta_{5}=115^{\circ }\right)$ and the QPSK signals from $\left(\theta_{6}=105^{\circ },\beta_{6}=95^{\circ }\right)$ , $\left(\theta_{7}=75^{\circ },\beta_{7}=40^{\circ }\right)$ and $\left(\theta_{8}=115^{\circ },\beta_{8}=70^{\circ }\right)$ . The SNR is 10 dB, and the number of snapshots is 500. Figure 2a,b indicates that the method can estimate and distinguish the 2D DOAs that are strictly noncircular and circular successfully.

### 5.2. Performance versus SNR

In this part, the performance of the proposed algorithm is studied with a varying SNR from −5 dB–15 dB. The number of snapshots is 500, and the number of Monte Carlo simulations is 500. Five BPSK signals and one QPSK signal are considered. The BPSK signals are from the directions $\left(\theta_{1}=65^{\circ },\;\beta_{1}=50^{\circ }\right)$ , $\left(\theta_{2}=90^{\circ },\;\beta_{2}=105^{\circ }\right)$ , $\left(\theta_{3}=50^{\circ },\;\beta_{3}=60^{\circ }\right)$ , $\left(\theta_{4}=125^{\circ },\;\beta_{4}=85^{\circ }\right)$ and $\left(\theta_{5}=30^{\circ },\;\beta_{5}=115^{\circ }\right)$ and the QPSK signals from $\left(\theta_{6}=105^{\circ },\;\beta_{6}=95^{\circ }\right)$ .

**Figure 3:**
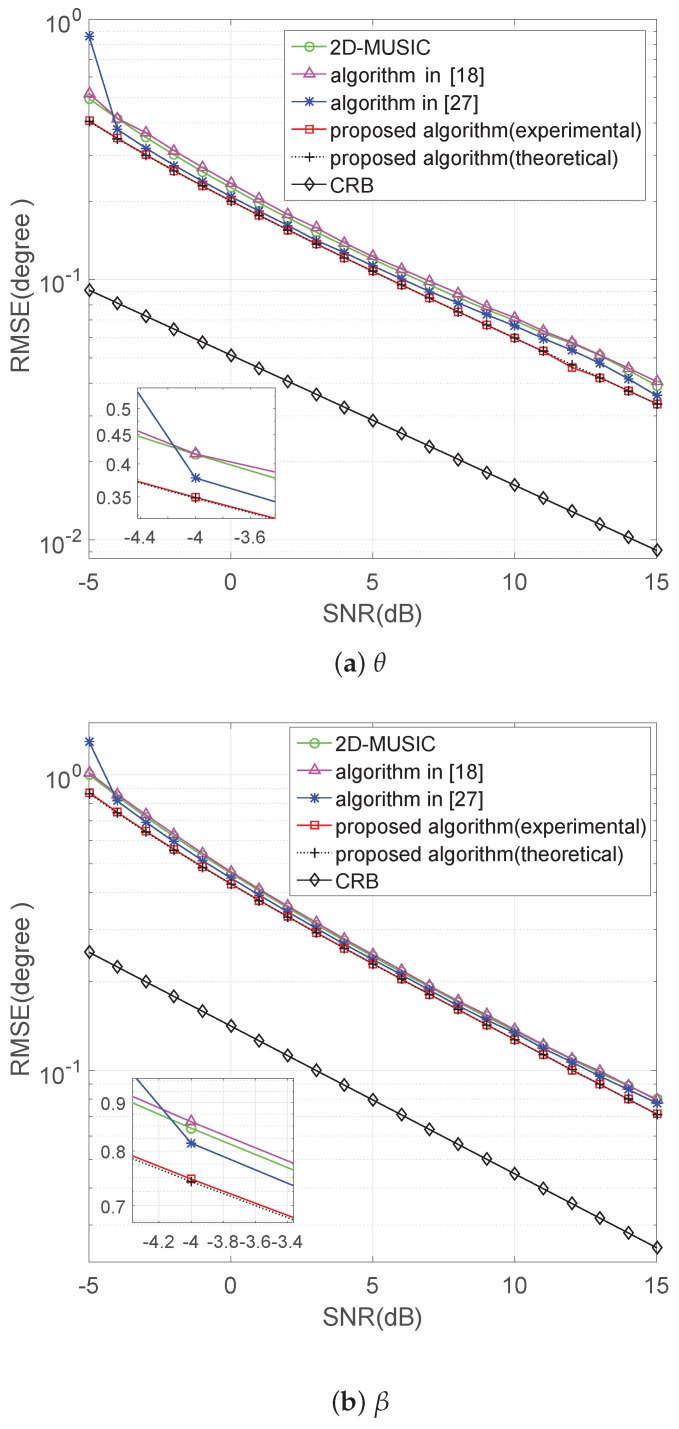
RMSE of versus SNR with the snapshots being 500.

The proposed algorithm in theoretical analysis and experimental results, the algorithm of 2D-MUSIC, the algorithm in [18], the algorithm in [27] and the deterministic CRB (Cramer–Rao bound) in [37], are compared in terms of RMSE. Figure 3a,b shows that the proposed method is steadily better than the other three algorithms, and the experimental values of the proposed algorithm overlap together with the theoretical error ones.

### 5.3. Performance versus Snapshots

**Figure 4:**
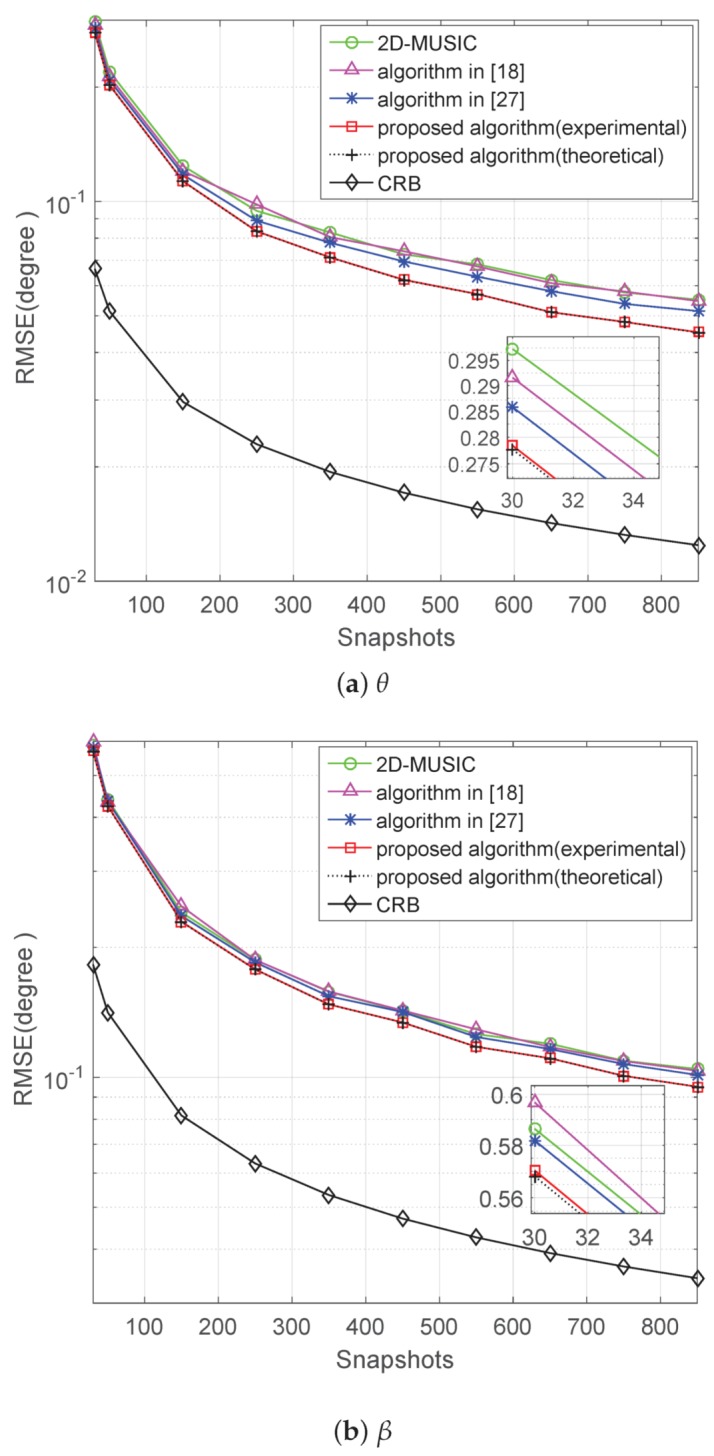
RMSE of versus snapshots when SNR = 10 dB.

We study the performance of the proposed algorithm with a varying snapshot number from 30–850. The SNR is fixed at 10 dB; the number of Monte Carlo simulations and incident signals are the same as in the second experiment. The RMSE results for angle estimation are shown in Figure 4a,b. The proposed method is steadily better than the other three algorithms, and the experimental values of the proposed algorithm overlap together with the theoretical error ones.

### 5.4. Performance versus the Number of Noncircular Mixed Signals

**Figure 5:**
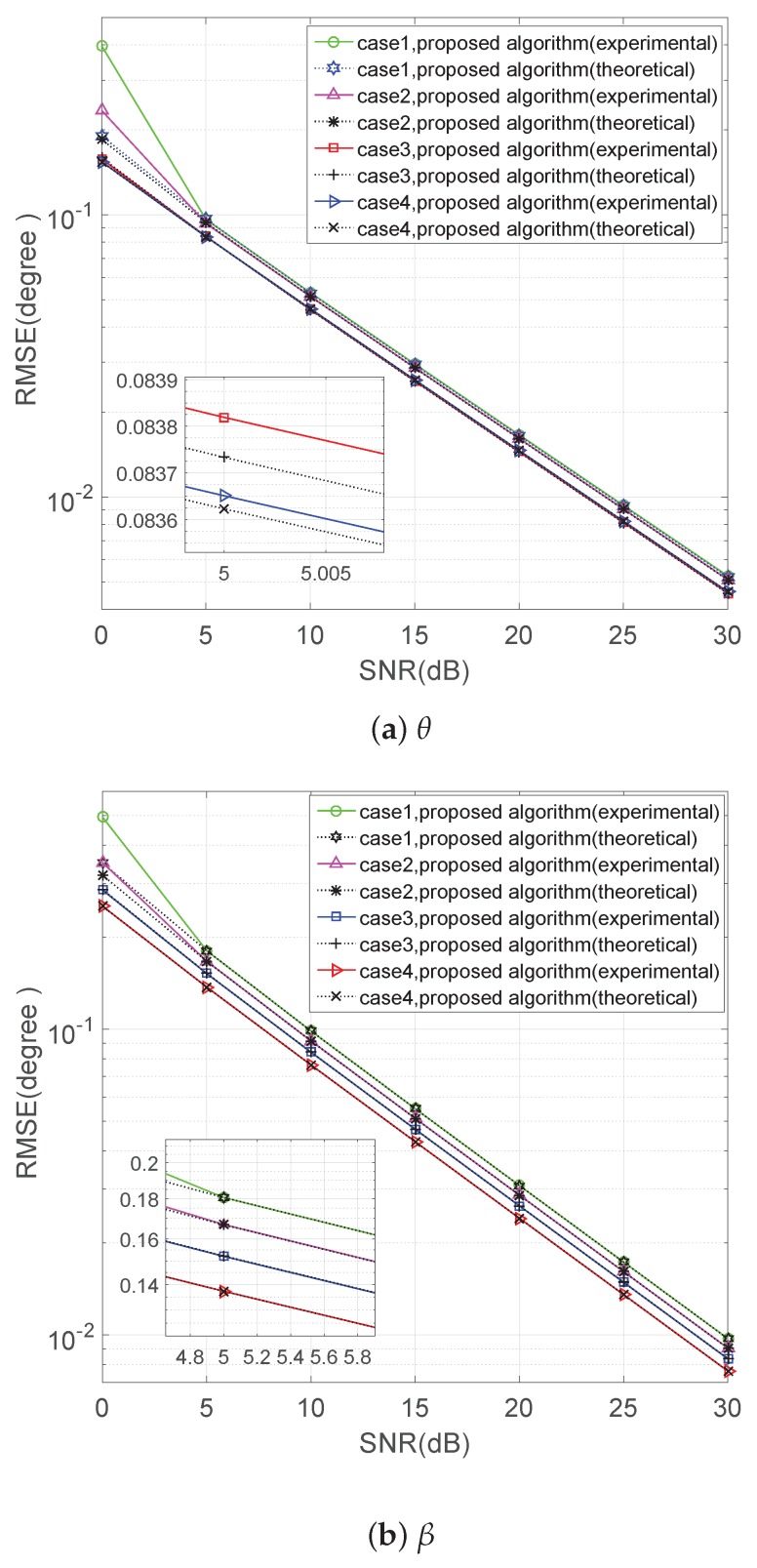
RMSE of versus SNR in four cases.

Finally, we consider the performance of the number of noncircular signals based on the proposed algorithm. The URAs have N=3 rows and M=6 columns; the SNRs vary form 0 dB–30 dB; the number of snapshots is 1200; and the number of Monte Carlo simulations is 1000. There are four uncorrelated signals from directions $\left(\theta_{1}=65^{\circ },\;\beta_{1}=50^{\circ }\right)$ , $\left(\theta_{2}=105^{\circ },\;\beta_{2}=95^{\circ }\right)$ , $\left(\theta_{3}=75^{\circ },\;\beta_{3}=40^{\circ }\right)$ and $\left(\theta_{4}=115^{\circ },\;\beta_{4}=70^{\circ }\right)$ , and the total number of sources remains unchanged. We consider cases about one, two, three and four BPSK signals, respectively. The results are shown in Figure 5a,b.

We can see that the RMSE of the experimental values of the proposed algorithm will overlap together with the theoretical error ones in a relatively high SNR, and the 2D DOA estimation performance of the proposed method improves from Case 1 to Case 4 because the dimension of the noise subspace has been extended by the increasing number of BPSK signals.

## 6. Conclusions

In this paper, a novel low-complexity 2D DOA estimation algorithm for mixed circular and non-circular signals has been proposed based on the URAs; besides, the theoretical error of the proposed algorithm is analyzed. As verified by the simulation results, the proposed method has a lower computational complexity and a better DOA estimation performance than the existing methods due to the usage of the noncircularity of the incoming signals.
